# A Cadaveric Case of an Accessory Cleido-Occipital Muscle Over the Common Trunk of the Supraclavicular Nerves

**DOI:** 10.7759/cureus.40982

**Published:** 2023-06-26

**Authors:** George K Paraskevas, Maria Tzika, Irene Asouhidou, Chrysanthos Chrysanthou, Elpida Apostolidi, Nectarios Galanis, Paraskevi Karamitsou, Alexandros Poutoglidis

**Affiliations:** 1 Department of Anatomy and Surgical Anatomy, Aristotle University of Thessaloniki, Thessaloniki, GRC; 2 Department of Otorhinolaryngology - Head and Neck Surgery, "G. Papanikolaou" General Hospital, Thessaloniki, GRC

**Keywords:** anatomical variations, entrapment syndrome, supraclavicular nerve, cleido-occipital muscle, muscle variation

## Abstract

The current study describes a case of an aberrant cleido-occipital muscle. In particular, this muscle was arising from the middle part of the clavicle, inserted into the medial part of the upper trapezius muscle, and crossed over the supraclavicular nerves with possible compression of them, especially during shoulder abduction. Knowledge of the muscular variability of the posterior cervical triangle is crucial for supraclavicular nerve entrapment syndrome diagnosis and treatment. The appearance of aberrant muscular fascicles may lead to misinterpretation of neck imaging, as well as difficulties during surgical procedures undertaken in the region.

## Introduction

The variant musculature of the posterior cervical triangle has been rarely described in the literature as compared to the shoulder region [1]. The presence of an accessory cleido-occipital muscle of the trapezius that originates from the medial and middle part of the clavicle and inserts into the cervical and occipital portion of the trapezius is a rare anatomical variation [2-10]. Such aberrant muscles may compress the common trunk and branches of the supraclavicular nerves, causing supraclavicular entrapment syndrome [7]. Muscle entrapment syndromes of the neck are well-described in the literature, with thoracic outlet syndrome being the most common [11]. The compression of the subclavian artery or the branchial plexus may cause significant problems in the ipsilateral arm movement and sensation [11]. Additionally, deviations from the normal development in anterior scalenus muscle formation and course often lead to compression of the above-mentioned neurovascular structures [11]. We present a rare cadaveric case of an abnormal muscular band that crosses over the supraclavicular nerve trunk, possibly compressing it during upper limb abduction.

## Case presentation

**Figure 1:**
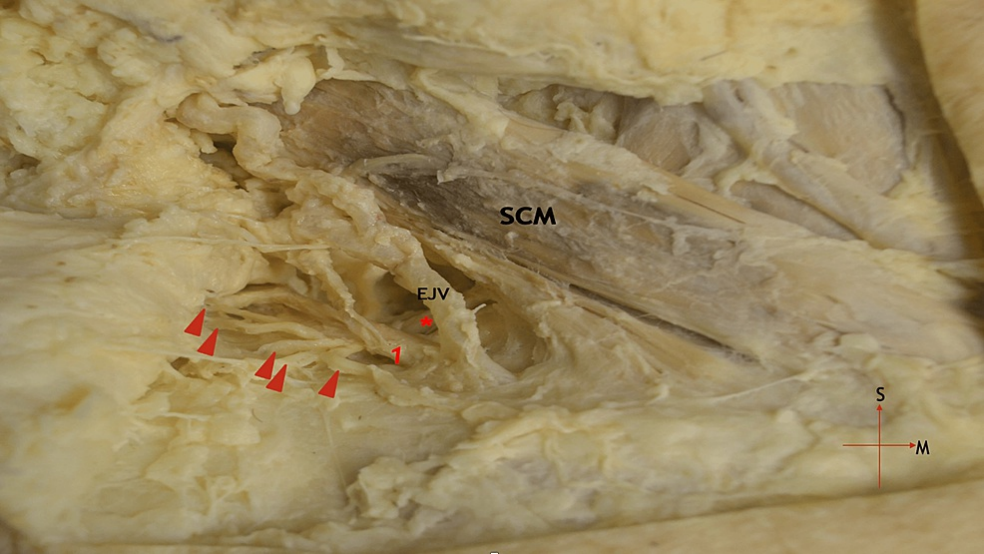
Lateral cervical region A right accessory cleido-occipital muscle is shown (1), running superficial to the common trunk (asterisk) of the supraclavicular nerves (arrowheads). SCM: sternocleidomastoid muscle; EJV: external jugular vein; M: medial; S: superior.

During a routine neck dissection of an 82-year-old male formalin-fixed cadaver for educational purposes at the Department of Anatomy and Surgical Anatomy of the School of Medicine of Aristotle University of Thessaloniki, an aberrant muscle was found in the supraclavicular region. A muscular band was noticed following the removal of the skin and subcutaneous fat of the right posterior cervical triangle, deep to the sternocleidomastoid muscle. It originated from the middle part of the clavicle, approximately 1.6 cm lateral to the sternocleidomastoid origin, and was inserted into the medial part of the upper trapezius muscle portion, blending with its muscle fibers. This accessory muscle crossed over the supraclavicular nerves’ common trunk and significant nerve compression was supposed to had been happening during shoulder abduction (Figure 1).

No other anomalies were present, and there was no history of neck surgery or trauma.

## Discussion

Both trapezius and sternocleidomastoid muscles derive from the brachial mesoderm and the adjacent myotomes. This common origin takes place at the occipital region, caudally to the last branchial arch [3]. Thus, embryological deviations from normal development have been commonly described in the literature. In fact, a supernumerary cleido-occipital head has been documented in approximately 1/3 of cases [2]. However, the presence of an aberrant distinct muscle band between the clavicle and trapezius muscle has been rarely documented and various nomenclature is found. In the present case, an abnormal muscle band was arising from the clavicle’s medial part and inserted into the upper trapezius portion, blending within its fibers. The described muscle was an anomalous cleido-occipitalis muscle. The term accessory cleido-occipital muscle of the trapezius is used for emphasizing the difference between this anomalous muscle band and the normal cleido-occipital portion of the trapezius muscle [3-5]. Sarikcioglu et al. [6] noted the presence of cleido-occipital muscle in 4.2% of cases.

The supraclavicular nerves are sensory branches of the cervical plexus that originate from the third and fourth ventral cervical rami and arise as a common trunk at the midpoint of the posterior sternocleidomastoid aspect (Erb’s point), along with the rest of the superficial cervical plexus branches. As it approaches the clavicle, the common trunk divides into a medial, an intermediate, and a lateral descending branch [7]. Entrapment of a supraclavicular nerve branch by an osseous clavicular tunnel was first presented in 1975 by Gelberman et al. [12]. Bony canals (transclavicular course of the nerve branches), fibrous bands, and aberrant musculature may cause compression of the supraclavicular branches [7].

In the described case, the aberrant muscle fibers coursed over the supraclavicular nerve common trunk and significant compression was supposed to be noticed during upper limb abduction, leading to a potential supraclavicular entrapment syndrome. Potential compression of the supraclavicular nerves due to an accessory cleido-occipital muscle of the trapezius has been previously reported in the literature [5]. Rahman and Yamadori [4] mentioned that the tendinous portion of the aberrant muscle that was detected in a specimen crossed over the supraclavicular nerves [4]. Furthermore, the cleidocervicalis muscle, which originates from the cervical vertebrae and inserts into the clavicle, and the supraclavicular proprius muscle attach to the clavicle [8-10].

## Conclusions

Awareness of the variant musculature of the posterior cervical triangle is essential for supraclavicular nerve entrapment syndrome diagnosis and treatment. The presence of accessory muscle fibers may lead to misdiagnosis during neck ultrasound and imaging, as well as to difficulties during surgical interventions in this anatomical area.
